# Supplementary material for: A rare PRIMER cell state in plant immunity
Source: Nature. 2025 Jan 8;638(8049):197–205. doi: 10.1038/s41586-024-08383-z (PMC11798839; doi:10.1038/s41586-024-08383-z)
Supplement: Supplementary file 1 — This file contains the Supplementary Discussion and supplementary references. [file 41586_2024_8383_MOESM1_ESM.pdf]

nature portfolio

https://doi.org/10.1038/s41586-024-08383-z

Supplementary information

A rare PRIMER cell state in plant immunity

In the format provided by the authors and unedited

Supplementary Information

Supplementary Discussion

1. Sampling strategy

In a study with a time-course experiment, any circadian effect is of major concern and needs to be addressed with careful experimental design and analyses. In this study, we harvested all samples at a similar time of day to mitigate any potential circadian effect. This was achieved by infiltrating pathogens into leaves at different times of the day. An alternative experimental design, as often used in bulk omics studies, would have been to infect plants simultaneously and harvest at different times with proper mock controls for each sampling time point. This was practically challenging for our single-cell analysis as we had to process as many samples as possible at the same time to run on the same 10x Genomics chip; and we had to use fresh tissues. This study aimed to capture diverse cell states in pathogen-infected leaves, which would include circadian effects. Our analysis did not focus on real-time course gene expression dynamics except for the pseudotime analysis to model an immune trajectory. The pseudotime trajectory inferred from the time series data (with potential circadian impact) formed a reasonable spatial gradient (Fig. 4f), implying that circadian effects did not significantly influence our pseudotime analysis. Therefore, we believe that the current experimental setup is sufficient to draw most of the main conclusions of our study.

2. Dimensionality reduction and clustering

Dimensionality reduction and clustering were performed independently on snRNA-seq and snATAC-seq data, with snRNA-seq data showing better resolution (Fig. 1b; Extended Data Fig. 1j); joint embedding and clustering of snRNA-seq and snATAC-seq showed a similar result with snRNA-seq alone (Extended Data Fig. 1k). Thus, we used the clustering based on snRNA-seq data for the rest of the analysis.

3. Immune cell populations

We systematically analyzed pseudobulk transcriptome of all 429 sub-clusters to select known and predicted immune-related genes (Extended Data Fig. 3b). These genes showed varying expression and patterns across the sub-clusters, suggesting that there are multiple immune

states in individual developmental cell types (Extended Data Fig. 3c). Mesophyll and epidermal cells tend to show stronger immune responses compared with vasculature cell types and cells with unknown identities (Extended Data Fig. 3c). Interestingly, cell populations from different cell types can take a similar immune cell state (Extended Data Fig. 3c), suggesting that some immune gene regulatory mechanisms are shared among multiple developmental cell types.

4. ACR-gene links

Cluster marker genes (1,774 genes) had more linked ACRs in a moderately distal region (2.5 kb to 5 kb from TSS) compared with non-marker genes (Extended Data Fig. 5c), implying the role of CREs moderately distal from the TSS in cell type/state-specific gene regulation, which is consistent with the observation in a previous study [1]. We also found that linked ACRs were slightly larger than non-linked ACRs (Extended Data Fig. 5d). The number of linkes per gene also varied depending on conditions; we found a higher number of links in pathogen-infected plants compared with non-infected plants (Extended Data Fig. 5e). We observed that the number of links explains how well the ATAC activity score (Extended Data Fig. 5f) correlates with gene expression (r^2=0.64; Extended Data Fig. 5g). Nevertheless, we found that maximum Pearson correlation coefficient values between each gene and linked peaks are a better predictor of correlations between gene expression and gene activity score (r^2=0.79; Extended Data Fig. 5h).

5. Discussion on non-linked pathogen-responsive genes

We found some immune genes were "not linked" and associated with constitutively opened chromatin, while their expression was tightly regulated upon pathogen attack (Extended Data Fig. 4f). The upstream regions of such non-linked immune genes were enriched with WRKY binding motifs (Extended Data Fig. 4g,h), implying that tight control of WRKY TFs, which can act as transcriptional activators or repressors, may regulate downstream genes. Constitutively accessible chromatin may facilitate the quick binding of TFs to the DNA for rapid induction of defense genes. It is also possible that other epigenetic modifications regulate non-linked genes. It has been shown that DNA methylation could inhibit the binding of WRKY TFs to DNA by steric hindrance[2,3]. It would be interesting to profile DNA methylation at the single-cell level to see if there is such gene regulation. Recently developed methods enable simultaneous single-cell profiling of transcriptome, chromatin accessibility, and DNA methylation from the same cells[4]. Such "single-cell triple-omics" technologies will further advance our understanding of gene regulation in plants.

6. Potential mechanisms of transcriptional suppression by GT-3A and its function

In a previous study, a trihelix transcription factor *BdTHX1* was shown to bind to an intronic region of a gene and was implied to transcriptionally suppress the gene. It is possible that a similar mechanism is at play for GT-3A. Intriguingly, we found a GT-3A binding motif in the introns of key SA regulators (*CPB60G* and *SARD1*) (Extended Data Fig. 9k**),** providing an interesting future opportunity to study the mechanisms of action of GT-3A in PRIMER cells. We found that PRIMER cell genes were not induced by DC3000 (Extended Data Fig. 9g), an immune-suppressive pathogen, suggesting that PRIMER cells are involved in immunity rather than susceptibility (e.g., pathogen exploiting the response).

7. How do PRIMER cells communicate with bystander cells?

Various signaling molecules, such as ROS, Ca, and DAMPs, may be involved in the communication between PRIMER and bystander cells. We found a number of PRIMER cell-specific genes with unknown functions, some of which may serve as mobile signals. Also, *ICS1* (a major SA biosynthesis gene) is induced in PRIMER cells (Extended Data Fig. 9h), suggesting that SA may be produced and translocated to neighboring cells via plasmodesmata or the apoplast. A previous study showed that SA accumulates in the apoplast and increases upon pathogen infection[5]. Understanding how the PRIMER cell functions and communicates with neighboring cells will be a new research topic in the field of molecular plant-microbe interaction, and we believe that our study provides a wealth of useful information.

8. Limitations of this study

Simultaneous spatial mapping of plant gene expression and bacterial colonization has great potential in elucidating interactions between plant cells and bacterial cells at the single-cell level (Extended Data Fig. 8g-i). In the future, time series MERFISH analysis of both immune-activating and -suppressive pathogen infection in replicates would provide a deeper understanding of how spatial relationships between plant and pathogen cells influence the responses of individual plant cells. However, it is challenging or impossible to fully capture such interactions on a 2D tissue section, and thus a 3D analysis of both plant genes and bacterial colonization is critical. Recently, we developed a technology called PHYTOMap[7], which can spatially map dozens of genes in 3D in whole-mount plant tissues. Such a method, combined

with our spatiotemporal atlas, will open a new avenue toward a comprehensive characterization of cell populations identified in this study. An outstanding question is “Are different immune cell states defined by pre-existing cell states with different immune potentials?” In addition to carefully assessing the spatial relationships between plant and pathogen cells in 3D, it is crucial to develop a new method to trace the molecular state before infection from different immune states after infection.

Supplementary Information References

1. Tu, X., Marand, A. P., Schmitz, R. J. & Zhong, S. A combinatorial indexing strategy for epigenomic profiling of plant single cells | bioRxiv. *bioRxiv* 2021.11.07.467612 (2021) doi:10.1101/2021.11.07.467612.
2. Charvin, M. *et al.* DNA methylation at a single cytosine embedded in the W-box cis-element repels binding of WRKY transcription factors through steric hindrance. *bioRxiv* 2022.09.26.509487 (2022) doi:10.1101/2022.09.26.509487.
3. O'Malley, R. C. *et al.* Cistrome and Epicistrome Features Shape the Regulatory DNA Landscape Resource Cistrome and Epicistrome Features Shape the Regulatory DNA Landscape. *Cell* **165**, 1280–1292 (2016).
4. Luo, C. *et al.* Single nucleus multi-omics identifies human cortical cell regulatory genome diversity. *Cell Genom* **2**, (2022).
5. Carviel, J. L. *et al.* Investigation of intercellular salicylic acid accumulation during compatible and incompatible arabidopsis-pseudomonas syringae interactions using a fast neutron-generated mutant allele of EDS5 identified by genetic mapping and whole-genome sequencing. *PLoS One* **9**, e88608 (2014).
6. Mine, A. *et al.* The Defense Phytohormone Signaling Network Enables Rapid, High-Amplitude Transcriptional Reprogramming during Effector-Triggered Immunity. *Plant Cell* **30**, 1199–1219 (2018).
7. Nobori, T., Oliva, M., Lister, R. & Ecker, J. R. PHYTOMap: Multiplexed single-cell 3D spatial gene expression analysis in plant tissue. *bioRxiv* 2022.07.28.501915 (2022) doi:10.1101/2022.07.28.501915.
