# Supplementary material for: A rare PRIMER cell state in plant immunity
Source: Nature. 2025 Jan 8;638(8049):197–205. doi: 10.1038/s41586-024-08383-z (PMC11798839; doi:10.1038/s41586-024-08383-z)
Supplement: Supplementary file 2 — Reporting Summary [file 41586_2024_8383_MOESM2_ESM.pdf]

nature portfolio

Corresponding author(s): Joseph R. Ecker

Last updated by author(s): Nov 7, 2024

Reporting Summary

Nature Portfolio wishes to improve the reproducibility of the work that we publish. This form provides structure for consistency and transparency in reporting. For further information on Nature Portfolio policies, see our Editorial Policies and the Editorial Policy Checklist.

Statistics

For all statistical analyses, confirm that the following items are present in the figure legend, table legend, main text, or Methods section.

n/a	Confirmed	
☐	☒	The exact sample size (*n*) for each experimental group/condition, given as a discrete number and unit of measurement
☐	☒	A statement on whether measurements were taken from distinct samples or whether the same sample was measured repeatedly
☐	☒	The statistical test(s) used AND whether they are one- or two-sided<br>*Only common tests should be described solely by name; describe more complex techniques in the Methods section.*
☒	☐	A description of all covariates tested
☐	☒	A description of any assumptions or corrections, such as tests of normality and adjustment for multiple comparisons
☐	☒	A full description of the statistical parameters including central tendency (e.g. means) or other basic estimates (e.g. regression coefficient) AND variation (e.g. standard deviation) or associated estimates of uncertainty (e.g. confidence intervals)
☐	☒	For null hypothesis testing, the test statistic (e.g. *F*, *t*, *r*) with confidence intervals, effect sizes, degrees of freedom and *P* value noted<br>*Give P values as exact values whenever suitable.*
☒	☐	For Bayesian analysis, information on the choice of priors and Markov chain Monte Carlo settings
☒	☐	For hierarchical and complex designs, identification of the appropriate level for tests and full reporting of outcomes
☐	☒	Estimates of effect sizes (e.g. Cohen's *d*, Pearson's *r*), indicating how they were calculated

Our web collection on statistics for biologists contains articles on many of the points above.

Software and code

Policy information about availability of computer code

Data collection	Illumina NovaSeq 6000, Vizgen MERSCOPE, BGI DNBSEQ-G400, GloMax Navigator Microplate Luminometer (Promega)
Data analysis	Seurat (v5.0.3), ggplot2 (v3.3.5), Signac (v1.12.9007), harmony (v0.1.0), org.Athaliana.eg.db (v0.1), chromVAR (1.14.0), STAR (v2.6.1b), fastp (v0.19.7), cellranger (v6.0.1), cellranger-arc (v2.0.0), cellpose (v2.1.1), scanpy (v1.9.1), scikit-learn (v1.1.2), spacexr (v2.2.1), Baysor (v0.5.2), scvi-tools (v1.0.2), clusterProfiler (v4.12.6), big-fish (v0.6.2), numpy (v1.24.4), opencv-python(v4.10.0.84), pygam (v0.9.1), edgeR (v4.2.1), limma (v3.60.6), ImageJ (v2.14.0), irlba (v2.3.5.1), qvalue (v2.36.0), Socrates (v0.0.9), scCustomize (v2.1.2).<br><br>Code availability:<br>The code to analyze snMultiome and MERFISH data is available at https://github.com/tnobori/snMultiome and https://github.com/amonell/Spatial_Plant_Pathogen_Atlas.

For manuscripts utilizing custom algorithms or software that are central to the research but not yet described in published literature, software must be made available to editors and reviewers. We strongly encourage code deposition in a community repository (e.g. GitHub). See the Nature Portfolio guidelines for submitting code & software for further information.

Data

Policy information about availability of data

All manuscripts must include a data availability statement. This statement should provide the following information, where applicable:

- Accession codes, unique identifiers, or web links for publicly available datasets
- A description of any restrictions on data availability
- For clinical datasets or third party data, please ensure that the statement adheres to our policy

All information supporting the conclusions are provided with the paper. The single-cell and bulk sequencing data generated in this study are deposited in the National Center for Biotechnology Information Gene Expression Omnibus database (accession no. GSE226826 and GSE248054). Reference genome, annotation, fully processed data for snMultiome analyses are available at neomorph.salk.edu/download/Nobori_etal_merfish. The MERFISH data are available at plantpathogenatlas.salk.edu. Genes targeted with MERFIHS are listed in Supplementary Table 1. Information of primers used in this study is provided in Supplementary Table 2. Source data are provided with this paper. Reference genome, annotation, fully processed data for snMultiome analyses are available at neomorph.salk.edu/download/Nobori_etal_merfish.

Human research participants

Policy information about studies involving human research participants and Sex and Gender in Research.

Reporting on sex and gender	N/A
Population characteristics	N/A
Recruitment	N/A
Ethics oversight	N/A

Note that full information on the approval of the study protocol must also be provided in the manuscript.

Field-specific reporting

Please select the one below that is the best fit for your research. If you are not sure, read the appropriate sections before making your selection.

☒ Life sciences ☐ Behavioural & social sciences ☐ Ecological, evolutionary & environmental sciences

For a reference copy of the document with all sections, see nature.com/documents/nr-reporting-summary-flat.pdf

Life sciences study design

All studies must disclose on these points even when the disclosure is negative.

Sample size	No statistical method was used to determine sample size. Instead, sample sizes were determined based on a combination of factors, including standard practices in plant biology research and the objectives of the experiments. For the pathogen growth assay, we selected sample sizes based on their effectiveness in previous similar studies in the field (such as PMID: 35545668, PMID: 37704725, and PMID: 35508659). For the snMultiome and MERFISH time course experiments, we primarily analyzed one replicate for each condition. Second replicates of snMultiome were performed for key conditions, which is a similar setup with a recent time course single-cell RNA-seq study (PMID: 36996230). In addition, we used two different pathogens known to induce similar responses and used matching conditions for independent snMultiome experiments, further ensuring the reproducibility of our data. Although the MERFISH experiments were performed with single replicates, the sample conditions match those of the snMultiome experiments, allowing cross-validation of conclusions from these orthogonal analyses. Therefore, we believe that the sample sizes used for snMultiome and MERFISH experiments are sufficient to support the conclusions of this study.
Data exclusions	No data was excluded for the analyses in this study.
Replication	For pathogen growth assays and bulk RNA-seq, three to four independent replicates were analyzed for each condition. Two independent replicates were analyzed for snMultiome for Mock and AvrRpt2 9 hr samples. No replicate was used for MERFISH time course analysis.<br><br>All attempts at replication reported were successful.
Randomization	Plants were grown in the same tray to ensure consistent environmental conditions. Individual plants were then randomly assigned to different treatment groups. This random allocation was employed to minimize selection bias and distribute any uncontrolled variables evenly across all treatment groups.
Blinding	In pathogen infection assays, certain treatments produce visible disease phenotypes, making it impossible to conceal the treatments applied. To minimize potential biases, we implemented consistent experimental protocols and standardized analysis pipelines for all samples.

Reporting for specific materials, systems and methods

We require information from authors about some types of materials, experimental systems and methods used in many studies. Here, indicate whether each material, system or method listed is relevant to your study. If you are not sure if a list item applies to your research, read the appropriate section before selecting a response.

Materials & experimental systems

n/a	Involved in the study
☒	☐ Antibodies
☒	☐ Eukaryotic cell lines
☒	☐ Palaeontology and archaeology
☒	☐ Animals and other organisms
☒	☐ Clinical data
☒	☐ Dual use research of concern

Methods

n/a	Involved in the study
☒	☐ ChIP-seq
☒	☐ Flow cytometry
☒	☐ MRI-based neuroimaging
